# Perioperative Pain Management Issues Unique to Older Adults Undergoing Surgery: A Narrative Review

**DOI:** 10.1097/AS9.0000000000000072

**Published:** 2021-07-01

**Authors:** Adam D. Shellito, Jill Q. Dworsky, Patrick J. Kirkland, Ronnie A. Rosenthal, Catherine A. Sarkisian, Clifford Y. Ko, Marcia M. Russell

**Affiliations:** From the * Department of Surgery, Harbor-UCLA Medical Center, Torrance, CA; † Department of Surgery, David Geffen School of Medicine at UCLA, Los Angeles, CA; ‡ VA Greater Los Angeles Healthcare System, Los Angeles, CA; § Department of Health Policy and Management, UCLA Fielding School of Public Health, Los Angeles, CA; ∥ Department of Surgery, Yale University School of Medicine, New Haven, CT; ¶ VA Connecticut Healthcare System, West Haven, CT; # Department of Geriatrics, David Geffen School of Medicine at UCLA and VA Greater Los Angeles Healthcare System, Los Angeles, CA.

**Keywords:** geriatric surgery, older adults, opioid sparing, pain management

## Abstract

**Introduction:**

The older population is growing and with this growth, there is a parallel rise in the operations performed on this vulnerable group. The perioperative pain management strategy for older adults is unique and requires a team-based approach for provision of high-quality surgical care.

**Methods:**

Literature search was performed using PubMed in addition to review of relevant protocols and guidelines from geriatric, surgical, and anesthesia societies. Systematic reviews and meta-analyses, randomized trials, observational studies, and society guidelines were summarized in this review.

**Management:**

The optimal approach to pain management for older adults undergoing surgery involves addressing all phases of perioperative care. Preoperative assessment of patients' cognitive function and presence of chronic pain may impact the pain management plan. Consideration should be also be given to intraoperative strategies to improve pain control and minimize both the dose and side effects from opioids (eg, regional anesthetic techniques). A multimodal postoperative pain management strategy minimizing opioids is crucial to providing adequate pain control while minimizing elderly-specific complications such as postoperative delirium and functional decline. Finally, pain management does not stop after the older adult patient leaves the hospital. Both discharge planning and postoperative clinic follow-up provide important opportunities for collaboration and intervention.

**Conclusions:**

An opioid-sparing pain management strategy for older adults can be accomplished with a comprehensive and collaborative interdisciplinary strategy addressing all phases of perioperative care.

## Abstract 2

Mini-abstract: This narrative review summarizes the unique perioperative pain management issues for older adults undergoing surgery. The focus of this comprehensive review is an opioid-sparing multimodality approach to ensure high quality and safe care for older adults throughout all phases of the perioperative period.

## INTRODUCTION

The population is aging and the number of older adults undergoing surgery is increasing.^1^ In addition to worse traditional surgical outcomes (ie, mortality, morbidity), older adults (age 65 years and older) are prone to geriatric-specific complications such postoperative delirium and functional decline.^2^ Aging affects every organ system and these physiologic changes need to be recognized and incorporated into geriatric-specific perioperative care.^3^

Pain management after surgery is becoming an increasingly important topic. This is particularly important in the older adult due to increased opioid sensitivity and risk of postoperative delirium.^4^ There is also growing attention to the use of opioids after surgery including recent research on excessive opioid prescriptions for common surgical procedures.^4,5^ The current momentum surrounding opioid overuse and abuse also highlights the need to minimize opioids in older adults undergoing surgery. While there are practice guidelines for pharmacologic management of persistent pain in older persons,^6^ there are no comprehensive reviews on perioperative pain management issues for older adults. The overarching goal of this narrative review is to highlight the unique perioperative pain management needs for older adults undergoing surgery with an emphasis on opioid-sparing and multimodal pain management techniques. An additional goal is to identify common pain management strategies for older adults that are relevant to and can be used across a range of surgical disciplines and operations.

## MATERIALS AND METHODS

Key areas for perioperative pain management in older adults were identified through a review of the literature across the following phases of perioperative care: preoperative, immediate preoperative, intraoperative, postoperative, and peri-/post-discharge. Example search terms included: “pain management,” “opioid-sparing,” “multimodal,” “geriatric,” “older adult,” and “elderly.” It should be noted that an opioid-sparing multimodality pain management protocol is one of the thirty standards required for implementation of the American College of Surgeons Geriatric Surgery Verification Program which was the impetus behind this review.^7^ We performed a nonsystematic literature review of the PubMed database in addition to a gray literature review of relevant protocols and guidelines from geriatric, surgical, and anesthesia societies.^8^

### Phases of Care

**FIGURE 1.**
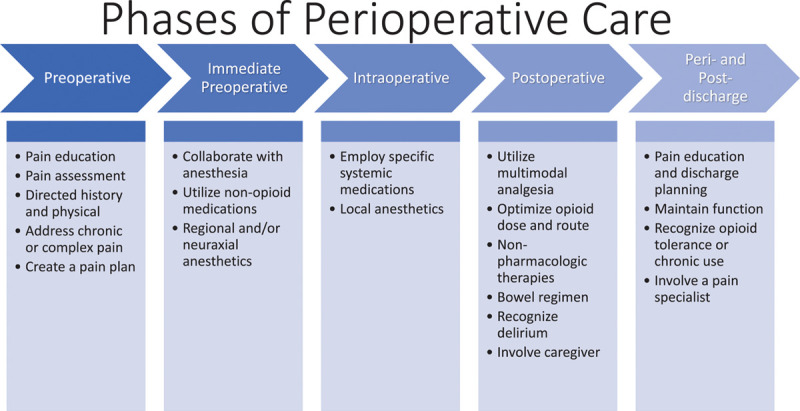
Pain management strategy overview for phase of perioperative care.

Pain management can be addressed across all phases of surgical care. Figure 1 provides a framework to approach perioperative pain management in the older adult. This systematic approach to management of pain by phase of surgical care will assist providers in caring for this unique population. The preoperative phase emphasizes the patients’ medical history and forms the basis of pain expectations through patient education. The immediate preoperative, intraoperative, and postoperative phases highlight the collaboration between provider teams, namely anesthesia and surgery, and focus on the multimodality approaches available to address diverse pain issues in this vulnerable population. The peri- and post-discharge phase again focuses on expectations with patient education as well as continued care with close follow-up to address any issues that may arise.

### Preoperative Phase

Arguably the most important part of an optimal pain management strategy, the preoperative phase of care sets the stage for successful pain management after surgery, particularly in the geriatric population.

Several meta-analyses have suggested that preoperative education can decrease postoperative pain and anxiety.^9,10^ The provider should explain the importance of pain control in the context of participating in postoperative recovery activities such as incentive spirometry and mobilization.^11,12^ The older adult should understand that they will have pain after surgery and that no combination of medications can make them completely pain free. Stronger pain medications (ie, opioids) should be reserved for pain that limits critical function or activities after surgery (ie, ambulation) as there are potential downstream side effects of opioid use in older adults such as postoperative delirium.

Discussing the anticipated pain assessment tools with the older adult prior to surgery is a key step that can be easily forgotten. Pain assessment, and thus pain treatment, is also more effective when the older adult understands the pain assessment tool. There are several assessment tools that are useful in measuring pain intensity in older adults, but some may be more useful depending on the patient’s sensory and cognitive abilities. For example, the Faces Pain Scale-Revised may be useful for more visually inclined patients as well as those with mild to moderate cognitive impairment, whereas the Numeric Rating Scale should be used in individuals who can understand a numerical scale and self-report their pain intensity.^13^ The Verbal Descriptor Scale (Pain Thermometer) can be used in patients with moderate to severe cognitive impairment, which is a common geriatric-specific comorbidity in older adults.^14^

For nonpharmacologic treatments, the provider should explain evidence-based options that are available postoperatively such as distraction strategies, guided imagery, or mindful breathing.^15^ Once the older adult has chosen preferred modalities, the provider should ensure that the older adult has the physical and mental abilities to utilize the chosen nonpharmacologic pain management. Physical and mental fatigue may interfere with the learning process, so for these modalities to be maximally effective, it is important for the older adult to learn them preoperatively when not yet afflicted by pain.^15,16^

Older adults and their caregivers should also be educated on the various pharmacologic treatment options. A simple explanation of how the drug works (eg, nonsteroidal anti-inflammatory drugs [NSAIDs]) should be discussed (Table 1). Misconceptions about pain treatments are common among older adults and can interfere with willingness to take certain analgesics, particularly fears regarding addiction and tolerance affecting patients’ willingness to take opioids.^16–18^ Infographics and visuals about pain management with easy to understand language can also be useful in the preoperative setting. A recent single-institution study by Angelo et al^19^ showed that a patient-friendly infographic in the preoperative area listing the common “do’s and don’ts” of pain medications was a useful adjunct to help decrease opioid consumption postoperatively.

**TABLE 1.** Common Pain Medications With MOA for Pain Relief

Medication	MOA
Acetaminophen	Nonspecific central COX inhibitor
NSAIDs	Inhibit COX enzymes, thereby reducing the synthesis of prostaglandins that are involved in inflammation
Nonselective COX inhibitors (eg, ibuprofen, naproxen, ketorolac)	
Selective COX2 inhibitors (eg, celecoxib)	
GABA analogs (eg, gabapentin and pregabalin)	Inhibits calcium influx, which reduces the excitability of dorsal horn neurons after tissue damage
Opioids (eg, morphine, hydrocodone, oxycodone, hydromorphone)	Mimic natural opioids and interact with mu, delta, or kappa opioid receptors

COX indicates cyclooxygenase; GABA, gamma-aminobutyric acid; MOA, mechanisms of action; NSAID, nonsteroidal anti-inflammatory drugs.

Every older adult should receive a directed pain history and physical examination during the preoperative period.^20,21^ The pain history should focus on previous experiences with painful conditions, pain strategies that have worked in the past, preferences for pain control and history of geriatric events (eg, delirium, fall).^22^ In addition, a history of abuse of other substances that may affect pain management (eg, benzodiazepines, alcohol) is an important component of the preoperative conversation.^20^ Although no studies have proven the efficacy of a preoperative pain history and physical exam, multiple anesthesia and pain societies agree that this information is essential in personalizing and optimizing perioperative pain management. This is particularly true in the aging adult who may have geriatric-specific comorbidities such as cognitive impairment or history of falls that influence the pain management strategy after surgery.^20,21^ In addition, assessment and documentation of the older adult’s cognitive function with a screening test such as the Mini-Cog can help identify patients with cognitive impairment or dementia who are at increased risk of postoperative delirium. Instruments like this can provide an important baseline reference of cognitive ability and guide choice of pain assessment tools as well as both pharmacologic (eg, minimization of opioids) and nonpharmacologic treatment options.^23,24^

In older adults with complex pain management needs (ie, chronic opioid use, opioid tolerance, or addiction), a pain specialist should be consulted for preoperative planning and postoperative management. When a designated pain specialist is unavailable or resources are limited, surgeons can collaborate with others that may have pain management experience such as anesthesiologists, physical medicine and rehabilitation physicians, palliative care physicians, or healthcare providers with geriatrics expertise (eg, geriatrician or hospitalist/internist/advance practice provider with geriatric training). An issue for this vulnerable population is balancing adequate pain treatment with risk of worsening addiction, as poorly treated pain can be a trigger for relapse as well as postoperative delirium that is commonly seen in the geriatric population.^20,25^ These patients may also be taking medications aimed at treating an opioid use disorder (eg, methadone, buprenorphine, naltrexone), further complicating their perioperative pain management.^26^

A preoperative pain management plan should be developed and individualized for each older adult given the unique comorbidities, previous experiences with pain, and patient preferences. In addition, it is also important to develop the plan in collaboration with the older adult’s family member/caregiver.^20^ An example of a pain management plan template is available from the American College of Surgeons Safe and Effective Pain Control After Surgery.^18^ The pain management plan should be centered around specific functional targets rather than a defined level of pain.^20,27^ Functional goals in the postoperative period should be both provider-driven (eg, early mobility or incentive spirometry) and patient-driven (eg, Sleeping comfortably). Importantly, these goals may impact other downstream outcomes in older adults such as postoperative delirium and functional decline.

### Immediate Preoperative Phase

The immediate preoperative phase is a crucial time period for collaboration with the anesthesiologists as well as implementing adjuncts for a successful pain management strategy. The surgeon should work with the anesthesiologist to review and incorporate the preoperative pain history and pain management plan to help close the loop between the preoperative and intraoperative phases of care.

Nonopioid adjuncts like acetaminophen, NSAIDs, and gamma-aminobutyric acid (GABA) analogs should be considered in the preoperative period for older adults. Table 2 depicts timing, example dosages for older adults, and side effects of common preoperative medications. The pathophysiology of preemptive analgesia is based on the idea of prevention of central sensitization, or the activation of central neurons by afferent signals that occur during surgery, leading to hypersensitivity postoperatively.^31^ A meta-analysis of 11 randomized controlled trials (RCTs) showed that a single dose of preoperative acetaminophen reduced early pain as well as postoperative opioid consumption.^32^ Acetaminophen is generally well tolerated, even in the older adult population.^33^ The available evidence supports a strong recommendation for preoperative acetaminophen use, delivered either orally or intravenously (IV). However, caution is advised when using acetaminophen near-maximal dosages, especially in the older adult demographic, as it can cause hepatotoxicity.

**TABLE 2.** Perioperative Medications, Timing, Dosages, and Potential Side Effects in Older Adults

Phase of Care	Medication	Timing	Example Dosing^28^	Maximum Dose	Side Effects	Special Considerations
Preoperative	Acetaminophen	Within 30–90 min of surgery	500 to 1000 mg IV or PO × 1 mg daily	3000 mg daily	Hepatotoxicity	Reduce maximum dose (2000 mg) if malnourished (<50 kg) or hepatic insufficiency^28^
	NSAIDs (eg, celecoxib)		200 mg PO celecoxib × 1 mg daily	Celecoxib: 400 mg daily	GI bleeding, renal dysfunction	Nonselective potentially inappropriate: risk of GI bleed, PUD, and AKI^29^
	GABA analogs (eg, gabapentin, pregabalin)		600 mg PO gabapentin OR 150 mg PO of pregabalin x 1 mg daily	Gabapentin: 2400 mg/d	Respiratory depression, sedation, dizziness	Potential drug-drug interaction with opioids^29^
				Pregabalin: 300 mg/d		
Intraoperative Peri-induction	Neuraxial/local	At time of induction or incision	Variable dose and route	Variable	Cardiotoxicity	Route specific to surgery
	Dexamethasone		0.11–0.2 mg/kg	8–25 mg per day	Blood glucose alterations	Anesthesia to dose
	Magnesium		30–50 mg/kg bolus followed by infusion	30–40 g per day	Hypotension	Anesthesia to dose
Postoperative	Acetaminophen	Initiate <24 h of surgery completion	500–1000 mg PO or IV q6h	3000 mg daily	Hepatotoxicity	Reduce maximum dose (2000 mg) if malnourished (<50 kg) or hepatic insufficiency
	NSAIDs		200 mg BID celecoxib OR 15 mg IV q6h ketorolac	Celecoxib: 400 mg daily	GI bleeding, renal dysfunction	Nonselective potentially inappropriate: risk of GI bleed, PUD, and AKI
				Ketorolac: 60 mg/d		
	GABA analogs		600 mg gabapentin OR 150 mg pregabalin q12h	See above	Respiratory depression, sedation, dizziness	Potential drug-drug interaction with opioids
	Topical patches		5% lidocaine patch (lidoderm) 12 h/d	NA	Local skin reaction	
	Opioids		Reduce by 25%–50% (eg, 2.5 mg oxycodone q6h)	Variable	Respiratory depression, sedation, tolerance, and addiction risks	Use equianalgesic table when converting^30^
			PCA when IV required (eg, morphine 1 mg q10min or hydromorphone 0.1 mg q10min)			

AKI indicates acute kidney injury; BID, twice daily; GABA, gamma-aminobutyric acid; GI, gastrointestinal; IV, intravenous; NSAIDs, nonsteroidal anti-inflammatory drugs; PCA, patient-controlled analgesia; PO, per oral; PUD, peptic ulcer disease; q10min, every 10 minutes; q12h, every 12 hours; q6h, every 6 hours.

Other preoperative premedication regimens include NSAIDs and GABA analogs (gabapentinoids). Clinical Practice Guidelines from the American Pain Society recommend the consideration of a preoperative dose of oral celecoxib, given that a number of RCTs have shown an associated reduction in postoperative opioid requirements and lower postoperative pain scores.^20,34,35^ A meta-analysis including 13 RCTs examining a single perioperative dose of ketorolac showed improvements in early pain at rest and a reduction in opioid consumption.^36^ However, it should be noted that noncyclooxygenase-selective NSAIDs (eg, ibuprofen, naproxen, ketorolac) were included on the 2019 Beers criteria of potentially inappropriate medications for older adults due to increased risk of gastrointestinal bleeding/peptic ulcer disease and acute kidney injury.^29^ However, the specific recommendation is to avoid chronic use of nonselective NSAIDs, unless alternatives are not effective and patient can take a gastroprotective agent (ie, proton pump inhibitor). Particularly high-risk groups for chronic NSAID use include older adults >75 years, those taking steroids or anticoagulants, those with a history of gastric or duodenal ulcers, and patients with chronic kidney disease stage 4 or higher (moderate quality of evidence, strong recommendation based on 2019 American Geriatrics Society [AGS] Beers Criteria).^29^ Thoughtful use of short-term NSAIDS may be appropriate in select older adults depending on comorbidities and desire to minimize opioids.

GABA analogs (eg, gabapentin, pregabalin) are another adjunct frequently used in the preoperative period. Several meta-analyses of RCTs have suggested beneficial effects of preoperative GABA analogs on postoperative pain.^37,38^ However, GABA analogs are associated with side effects that may disproportionately affect older adults (eg, respiratory depression, sedation, dizziness). Furthermore, the combination of opioids and GABA analogs was added to the 2019 AGS Beers Criteria as a potentially inappropriate medication combination for older adults, due to a drug-drug interaction increasing the risk of opioid overdose and sedation-related adverse events. However, the recommendation includes an exception when transitioning from opioid therapy to gabapentin or pregabalin or when using gabapentinoids to reduce opioid dosages (moderate quality of evidence, strong recommendation based on 2019 AGS Beers Criteria).^29^ Given the recommendation from the 2019 AGS Beers Criteria regarding GABA analogs, thoughtful use of GABA analogs can be considered as part of enhanced recovery after surgery pathways tailored specifically to older adults.

Regional or neuraxial anesthetic techniques can be safely used in older adults and should be considered if appropriate for the operation.^20,21,39^ Examples of specific operations for which regional or neuraxial anesthetic techniques are commonly used include the following: thoracotomy (eg, paravertebral block), colectomy (eg, epidural or transversus abdominis plane blocks), and extremity operations (eg, site-specific regional blocks for orthopedic or vascular surgery).^20^ Several meta-analyses and systematic reviews across a variety of surgical procedures suggest that regional and neuraxial techniques are associated with decreased pain scores and decreased need for analgesic medication postoperatively.^40–43^ Regional anesthesia is associated with earlier ambulation, earlier return of bowel function and improved mental status.^28^ Additionally, regional techniques have minimal effect on hemodynamics and do not cause urinary retention, a common complication following any surgical procedure in the older adult population. Neuraxial techniques may allow for reduced opioid dosing and thus can benefit the older adult in terms of cognitive function, improved mobility, and prevention of pulmonary complications.^17^ Increased attention to use of regional or neuraxial techniques can be an important component of an opioid-sparing multimodality pain management protocol in older adults with the goal of minimizing downstream outcomes like postoperative delirium and functional decline.

### Intraoperative Phase

There are limited but important interventions that can occur during the intraoperative phase of care in the geriatric population that necessitate collaboration and communication with the anesthesia team. Local anesthetic should be considered for intraoperative administration via at least one method (ie, regional/neuraxial or subcutaneous). Multiple systematic reviews of RCTs have found improved pain scores and greater pain relief when neuraxial opioid is combined with local anesthetic, compared with neuraxial opioid alone.^44,45^ American Pain Society Guidelines recommend that providers consider surgical site-specific local anesthetic infiltration for select surgical procedures. Although there are no RCTs showing the efficacy of subcutaneous local anesthetic, there is a low risk of harm when using appropriate doses and should be considered for a wide variety of surgical procedures in older adults.^46^ One example of local anesthetic infiltration is continuous local anesthetic infusion catheters (ie, ON-Q pumps) that provide nonopioid pain relief delivered directly to a surgical site for up to 5 days. These pumps have been shown to be useful in a variety commonly performed surgeries in the geriatric population, for example, orthopedic procedures and open abdominal operations such as laparotomy for colorectal resections.^47,48^ While there is no data specific to use of local anesthetic in older adults, it is a low-risk intervention that may reduce opioid use and improve mobility, therefore minimizing the risk of postoperative delirium and functional decline.

Several drugs, including dexamethasone and magnesium, delivered either before induction of anesthesia, at induction or intraoperatively have shown to be important adjuncts in preventive analgesia, a central tenet for optimal pain management after surgery in older adults. Dexamethasone, which is frequently given to decrease postoperative nausea and vomiting, has also been shown to have analgesic and opioid-sparing effects.^49^ Magnesium, which serves as an antagonist to N-methyl-d-aspartate glutamate receptors and can therefore alter pain response, has been used for many decades in an attempt to reduce postoperative pain.^50,51^ Meta-analyses of RCTs examining both of these medications in a variety of procedures have shown promising results with low rates of adverse effects.^49,52,53^ The dosages of these medications are weight based, and should be administered by the anesthesiologist (Table 2).

IV ketamine is another medication that could be considered for intraoperative administration (weak recommendation, moderate quality of evidence per the 2019 AGS Beers Criteria).^20^ A meta-analysis of RCTs in adults of all ages showed that intraoperative ketamine may offer protection against postoperative cognitive dysfunction, which is particularly relevant to the older adult population.^54^ However, a recent randomized trial in older adults showed that while ketamine did not have increased rates of delirium, it also did not affect postoperative opioid requirements or pain and can cause negative experiences like hallucinations or nightmares.^55^ In summary, local anesthetic, dexamethasone, magnesium, and ketamine are intraoperative medications that can be considered for use in older adults undergoing surgery with the goal of optimizing pain control while minimizing opioid use.

### Postoperative Phase

Pain control in the older adult requires adequate treatment of pain while simultaneously avoiding over treatment with opioids. Uncontrolled pain is more common in older adults and is also a precipitating factor for postoperative delirium.^56^ However, the risk of harmful side effects of opioids in older adults (eg, triggering postoperative delirium) must be balanced with appropriate pain control to facilitate mobility and minimize postoperative functional decline after surgery.^17,28^

**FIGURE 2.**
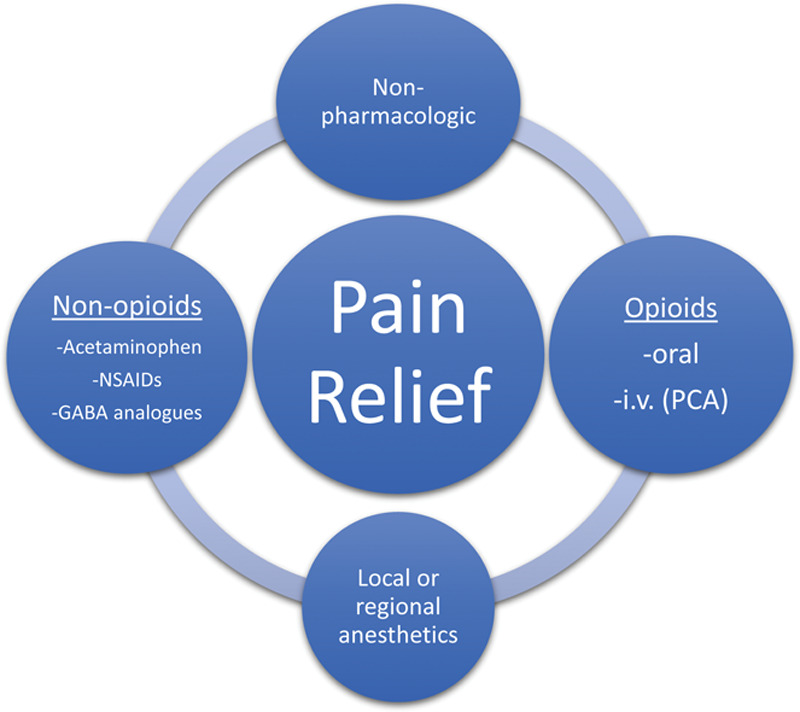
Postoperative multimodal pain strategy for successful pain relief in the older adult. GABA indicates gamma-aminobutyric acid; i.v., intravenous; NSAIDs, nonsteroidal anti-inflammatory drugs; PCA, patient-controlled analgesia.

The cornerstone of the postoperative phase of care in the older adult population involves a multimodal pain management strategy, employing medications with different mechanisms as well as nonpharmacologic pain relief therapies (Fig. 2) with the goal of minimizing opioid use.^21,52^ RCTs with numerous combinations of drugs acting at different receptors, medications administered via different techniques, and nonpharmacologic modalities have shown that these strategies result in a reduction in postoperative pain, a reduction in the consumption of any one analgesic drug, and in many cases a reduction in the consumption of opioid analgesics.^20,57,58^ Older adults have increased sensitivity to opioid and nonopioid medications, so this population may have the most to benefit from multimodal pain regimens.^28^ However, the choice of a specific multimodal pain regimen should be individualized to both the older adult and type of surgery. Particular attention must be paid to drug-drug interactions and potential medication side effects when designing a multimodal pain plan for older adults, as this population is at increased risk for side effects from polypharmacy and postoperative delirium.

There are multiple medications that can be scheduled around-the-clock to help decrease the need for opioids in the older adult population. Acetaminophen is an excellent drug to use in older adults, as it does not cause any gastrointestinal disturbance and has low risk for toxicity except in those with severely impaired liver function.^28^ As discussed in the immediate preoperative section, the use of NSAIDs in the older adult population is controversial. The optimal duration for administration of NSAIDs to avoid adverse side effects in older adults has not been directly studied so it is recommended that they be used with caution and for the shortest duration possible to minimize the risk of side effects.^59^ Similar to regional strategies addressed in the preoperative phases, nerve blocks (either single-shot or continuous) can be safely employed postoperatively in the older adult population for certain operations including knee replacement therapy^60^ or in the setting of hip fractures in the elderly.^61^ In addition, patient-controlled epidural analgesia is an effective method to consider for a variety of procedures including laparotomy for general surgery or gynecologic operations. The exact patient-controlled epidural analgesia regimen should be tailored to the older adult to avoid adverse events such as hypotension or inadequate pain control, which are commonly seen in this cohort of older adult patients.^62^ Topical anesthetic agents (eg, lidocaine patch, vapocoolant anesthetic sprays) can also be considered for postoperative administration although these have variable data to support routine use.^6,63,64^

The evidence-based nonpharmacologic pain management therapies learned and practiced preoperatively (eg, guided imagery, mindful breathing) should be implemented as early as possible in the postoperative setting in the older adult population. Overall, studies of these modalities have shown positive effects on postoperative pain, anxiety, and analgesic use, with no significant harm.^20^ Other therapies that do not require preoperative learning can be introduced by nursing staff (eg, superficial massage, repositioning, superficial heat or cold, and vibration).

When treating older adults with opioids, careful dose titration is essential. The ASA guidelines include a short section on geriatric patients where it is acknowledged that vigilant dose titration is necessary due to altered physiology, comorbidities, and the concurrent use of other medications.^21^ Many are familiar with the adage “start low and go slow,” and it is generally well-accepted by the geriatrics community that a dose reduction of 25%–50% of the normal adult dose of opioids is a reasonable starting point (Table 2).^17,28^ For example, a more appropriate starting dose of oxycodone in an older adult would be 2.5 mg rather than 5 mg. In addition, when increasing the dose of opioids, an increase of 25% is reasonable until there is a 50% reduction in the patient’s pain rating or the patient reports satisfactory pain relief.^65^

When opioids are used in the older adult population, the oral route should be used over the IV route whenever possible.^20,66^ IV opioids have the highest risk of adverse events such as sedation, respiratory depression, cognitive impairment, and postoperative delirium.^28^ However, there are many instances where IV opioids are necessary, such as in the immediate postoperative period or if patients must be nil per os after abdominal surgery. When IV opioids are necessary, patient-controlled analgesia is the gold standard in terms of successful analgesia as well as patient satisfaction (see Table 2, eg, starting doses).^28^ However, this modality should not be used in older adults with severe dementia or delirium who are unable to understand how to push the button as this may result in inadequate analgesia.^17^ Intramuscular administration of medications should not be used in the older adults due to increased pain and unreliable absorption.^20,21,67^ Older adults have muscle wasting and decreased fatty tissue that leads to slower intramuscular absorption and potentially delayed or prolonged effects, which can subsequently lead to accumulated toxicity with repeated injections.^17^ Lastly, if changing to a new opioid agent or different route of administration in the older adult patient, the new dose should be calculated with an equianalgesic table^30^ and the dose should be lowered by 25%–50% of oral morphine equivalents.^65^

**FIGURE 3.**
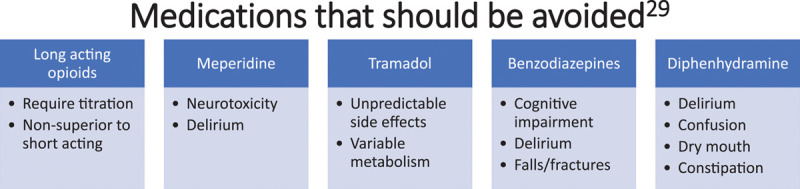
Medications to be avoided in older adult based on 2019 Beers Criteria.^29^ GABA indicates gamma-aminobutyric acid.

While opioids can be a critical part of the postoperative pain management plan, there are certain opioids that should be avoided in older adults (Fig. 3). For example, long-acting opioids (eg, MS Contin, OxyContin, fentanyl patch) should be avoided for opioid-naive older adults as there is no evidence that they provide better pain control than short-acting opioids.^20,68^ Meperidine is another potentially inappropriate medication for older adults as it has a higher risk of neurotoxicity, including postoperative delirium, compared with other opioids (moderate quality of evidence, strong recommendation based on 2019 AGS Beers Criteria).^29^ Lastly, tramadol should be used with caution due to unpredictable effects^69^ and concern for increased risk of delirium.^28,70^ Finally, tramadol was recently included in the 2019 AGS Beers Criteria^29^ and has also been referred to as “tramadon’t” in the geriatrics community due to the variable metabolism and unfavorable side effect profile in the older adult.^71^

Older adults are more sensitive to opioids and their adverse effects, including constipation.^28,72^ Analgesic plans for all older adults postoperatively should include a prophylactic pharmacologic bowel regimen.^39^ An optimal prophylactic pharmacologic bowel regimen should include more than just a stool softener such as a bulking agent (eg, fiber), stimulant laxative (eg, Bisacodyl, Senna) or osmotic laxative (eg, polyethylene glycol).^73^

Accurate assessment of pain in the older patient with impaired cognition can be difficult, and unfortunately, clinicians often under-treat pain in this patient population.^72^ The cognitively impaired older adult may have trouble verbally communicating pain levels, so behavioral indicators can act as an indirect measure of pain.^73^ Examples include nonverbal cues/behaviors such as restlessness and agitation, vocalizations such as groaning or moaning, facial expressions such as brow lowering with jaw drop or mouth open, and a change in usual behavior such as new onset of confusion or aggression.^73^ Appropriate pain assessment tools should be used to guide interventions. Examples of appropriate pain assessment tools in this population include the Checklist of Nonverbal Pain Indicators, Pain Assessment in Advanced Dementia, and the Critical Care Pain Observation Tool.^74–76^ Frequent assessments at regular intervals after surgery help determine adequacy of pain relief, monitor progress toward functional goals, and recognize early signs of postoperative delirium, which is of utmost importance in the older adult patient. Postoperative delirium is associated with increased rates of postoperative mortality, morbidity and need for discharge to skilled nursing facility as well as increased healthcare costs.^77^

Postoperative delirium is the most common complication in older adults and rates can vary by type of operation. Delirium deserves special attention as this complication disproportionately affects older adults and profoundly affects their recovery after surgery.^78,79^ Importantly, up to 40% of cases of delirium are preventable.^80^ Prophylactic strategies to prevent delirium include early mobilization and sensory enhancement by means of ensuring glasses, hearing aids or listening amplifiers are available to those who need them. Additionally, frequent reorientation and appropriate sleep hygiene are crucial in this population postoperatively.

Once a delirium episode has been identified, further workup is needed to identify potential underlying causes for the delirium such as infection, electrolyte abnormalities, environmental contributors, and medications. Pharmacotherapy for management of delirium may be initiated if nonpharmacologic methods or management of precipitating conditions are not effective. The AGS recommends against the routine use of benzodiazepines as a first-line agent for the treatment of postoperative delirium, even when complicated by agitation or threats of harm to self/others in the elderly. In the severely agitated elderly patient experiencing delirium, the recommended treatment is an antipsychotic at the lowest effective dose for the shortest possible duration after all nonpharmacologic interventions have been exhausted.^81^ Practitioners should exhibit caution in prescribing antipsychotics for delirium and reserve pharmacotherapy for the treatment of older adults who are agitated or threatening substantial harm to self or others. Recent data shows that low-dose haloperidol (<3.0 mg/d) is equally efficacious as the atypical antipsychotics olanzapine and risperidone in the treatment of delirium.^82^ High-dose haloperidol (>4.5 mg/d) is associated with extrapyramidal adverse effects (parkinsonism) and should be avoided in the older age patient population.^83^

### Peri-and Post-Discharge Phase

The transition home after surgery can be challenging for patients, and this is especially true for older adults and their caregivers. With older adults, it is important to involve family members/caregivers in discharge education, which includes medication education. Topics specific to pain management that should be addressed include expectations for pain, tapering of opioid medications and how to dispose of unused opioids. In addition, recognition of problems related to pain management including constipation, functional decline and postoperative delirium should be reviewed. Easy to follow instructions and patient infographics for pain after surgery can be used to reinforce the importance of these topics.^19^ Equally important, the older adult should be counseled on mobility exercises (eg, daily walking program) to perform after discharge to prevent functional decline and must be informed that these may also be associated with some pain and discomfort.^84^ At the same time, it is important that the older adult treats his or her pain adequately so as to not impair function. A pain management plan including a list of instructions for the older adult and family member/caregiver should be reviewed with the older adult and caregiver prior to discharge.^18^

## CONCLUSIONS

The pain management needs and strategy are unique for older adults undergoing surgery. While pain management often needs to be tailored to specific operations, the current narrative review aimed to highlight important themes for older adults that can be applied across a myriad of surgical disciplines. A multidisciplinary approach spanning all phases of perioperative care is necessary to provide a safe and effective pain management plan for older adults undergoing surgery. In addition, it should be noted that opioid-sparing, multimodality pain management is one of the hospital standards for the recently launched American College of Surgeons Geriatric Surgery Verification Program.^7^

The overarching goal of improving pain management in older adults is to optimize pain control while minimizing the side effects from opioids that can contribute to other downstream outcomes in this vulnerable population such as postoperative delirium and functional decline. A summary of the pain management issues unique to older adults are outlined by phase of care below.

### Preoperative Phase

This phase is hallmarked by a thorough history focusing on prior issues with pain and geriatric-specific issues unique to the older adults such as prior falls or delirium. Additionally, there is an emphasis on education of both the older adult and their family/caregiver about expectations for pain after surgery and the importance of meeting functional goals such as ambulation.

### Immediate Preoperative Phase

This phase is highlighted by a structured coordination between the operative and anesthesiology teams. Preoperative use of nonopioid medications such as acetaminophen, NSAIDs, and GABA analogs should be considered based on the older adult’s other medical problems. In addition, an emphasis on incorporation of regional or neuraxial anesthetic techniques are important to minimize opioids and improve pain control.

### Intraoperative Phase

This phase focuses on optimizing patient’s pain control prior to awakening from anesthesia as well as minimizing narcotics. Strong consideration should be given to judicious use of local anesthetics as well as continuation of neuraxial/regional anesthesia. Medications such as dexamethasone, magnesium, and ketamine may be used as adjuncts for an opioid-sparing multimodal pain plan.

### Postoperative Phase

This phase proves to be a delicate balance between adequate pain control using a multimodality approach and avoidance of side effects seen with common opioid pain medications. Minimization of opioids can be accomplished by use of nonpharmacologic methods for pain control, nonopioid medications, and use of smaller doses of opioids in this vulnerable population. The relationship between pain and postoperative delirium is reviewed including a brief overview of delirium prevention, workup, and treatment.

### Peri-and Post-Discharge Phase

This phase again emphasizes pain expectations and communication between the provider, patient, and importantly, the patient’s family/caretakers regarding management of pain after discharge from the hospital. The goal is to return patients to their prior level of function while minimizing complications related to pain management (eg, constipation, postoperative delirium, functional decline).

## ACKNOWLEDGMENTS

A.D.S. participated in research design, writing of the article, and in data analysis. J.Q.D. and M.M.R. participated in research design, writing of the article, performance of the research, and in data analysis. P.J.K. participated in writing of the article and in data analysis. R.A.R., C.A.S., and C.Y.K. participated in research design, writing of the article, and in data analysis.
